# Advanced glycation end products in children with type 1 diabetes and early reduced diastolic heart function

**DOI:** 10.1186/s12872-017-0551-0

**Published:** 2017-05-25

**Authors:** Leif Brunvand, Martin Heier, Cathrine Brunborg, Kristian F. Hanssen, Drude Fugelseth, Knut Haakon Stensaeth, Knut Dahl-Jørgensen, Hanna Dis Margeirsdottir

**Affiliations:** 1 Department of Pediatrics, Oslo University Hospital, Oslo, Norway; 2 Department of Neonatal Intensive Care, Oslo University Hospital, Ullevål, Oslo, Norway; 3 Oslo Diabetes Research Centre, Oslo, Norway; 4 Oslo Centre for Biostatistics and Epidemiology, Research Support Services, Oslo University Hospital, Oslo, Norway; 5 Institute of Clinical Medicine, Faculty of Medicine, University of Oslo, Oslo, Norway; 6 Department of Radiology and Nuclear Medicine, St. Olavs Hospital, and Institute of Circulation and Medical Imaging, Norwegian University of Science and Technology, Trondheim, Norway; 7 The Department of Paediatric and Adolescent Medicine, Akershus University Hospital, Lørenskog, Norway

## Abstract

**Background:**

Reduced diastolic function is an early sign of diabetes cardiomyopathy in adults and is associated with elevated levels of HbA1c and advanced glycation end products (AGEs).

**Objective:**

To assess the associations between early reduced diastolic function and elevated levels of HbA1c and AGEs in children and adolescents with type 1 diabetes (T1D).

**Methods:**

One hundred fourty six T1D patients (age 8–18 years) without known diabetic complications were examined with tissue Doppler imaging and stratified into two groups according to diastolic function. A clinical examination and ultrasound of the common carotid arteries were performed. Methylglyoxal-derived hydroimidazolone-1 (MG-H1) was measured by immunoassay.

**Results:**

At inclusion, 36 (25%) participants were stratified into a low diastolic function group (E’/A’-ratio < 2.0). Compared to the rest of the T1D children, these participants had higher body mass index (BMI), 22.8 (SD = 4.0) vs. 20.1 (SD = 3.4) kg/m^2^, *p* < 0.001, higher systolic blood pressure 104.2 (SD = 8.7) vs. 99.7 (SD = 9.3) mmHg, *p* = 0.010, and higher diastolic blood pressure, 63.6 (SD = 8.3) vs. 59.9 (SD = 7.9) mmHg, *p* = 0.016. The distensibility coefficient was lower, 0.035 (SD = 0.010) vs. 0.042 (SD = 0.02) kPa^−1^, *p* = 0.013, Young’s modulus higher, 429 (SD = 106) vs. 365 (SD = 143), *p* = 0.009, and MG-H1 higher, 163.9 (SD = 39.2) vs. 150.3 (SD = 33.4) U/ml, *p* = 0.046. There was no difference in carotid intima-media thickness between the groups. There were no associations between reduced diastolic function and years from diagnosis, HBA1c, mean HBA1c, CRP or calculated glycemic burden. Logistic regression analysis showed that BMI was an independent risk factor for E’/A’-ratio as well as a non-significant, but relatively large effect size for MG-H1, indicating a possible role for AGEs.

**Conclusions:**

Early signs of reduced diastolic function in children and adolescents with T1D had higher BMI, but not higher HbA1c. They also had elevated serum levels of the advanced glycation end product MG-H1, higher blood pressure and increased stiffness of the common carotid artery, but these associations did not reach statistical significance when tested in a logistic regression model.

## Background

Diabetic cardiomyopathy is associated with increased stiffness in the atrial and ventricular walls of the heart, and also with arterial stiffness [1]. Increased stiffness of the left ventricle leads to a reduced peak early (E’), and increased peak late (A’) diastolic velocity of the mitral ring [2]. The late peak velocity reflects the contraction of the left atrium. When diastolic function is impaired, the peak early blood velocity (E) across the mitral valve (MV) will initially be reduced [3]. Later, in more advanced diastolic dysfunction, the pressure in the left atrium increases and the blood flow across the valve will increase (pseudo-normalization). Consequently, the E/E’ ratio will increase with more severe diastolic dysfunction. E’, A’ and E can easily be measured with Doppler techniques in a standard echocardiographic examination [3]. Adult patients with T1D have a considerable prevalence of diabetic cardiomyopathy, but some studies have also demonstrated cardiac changes in diabetic children and young adults [4–6].

Glucose residues or metabolites of glucose can react non-enzymatically with proteins to form advanced glycation end products (AGEs) [7]. Such modifications may impair the function of key proteins [8]. Some AGEs also have the ability to create cross-links between long-lived structural proteins like collagen, contributing to the development of arterial stiffness [9]. Production of AGEs occurs in healthy subjects as well, but the formation is markedly accelerated in diabetes patients due to the increased availability of glucose [10]. Elevated levels of AGEs are considered important pathogenic factors in the development of diabetic cardiomyopathy [11]. This is supported by studies showing associations between AGEs and both arterial stiffness and impaired left ventricular function [12–16]. Furthermore, anti-AGE treatment improved collagen solubility and attenuated diabetes induced cardiac disease in rodents [17–19]. Various AGEs exist in different tissues, but methylglyoxal-derived hydroimidazolone-1 (MG-H1) is the most abundant AGE in human plasma [20]. We have previously shown that increased levels of MG-H1 in children with T1D are associated with early signs of atherosclerosis [21]. To our knowledge, the influence of MG-H1 as a risk factor for diastolic dysfunction in the early stages of cardiomyopathy in children and adolescents with T1D has not previously been studied.

## Methods

### Research design and study population

In 2006, all children and adolescents with T1D aged 8–18 years enrolled in the Norwegian Childhood Diabetes Registry (NCDR) and living in the South-East Health Region of Norway (*n* = 800) were invited to participate in the Atherosclerosis and Childhood Diabetes study at Oslo University Hospital, Ullevål, focusing on risk factors and early signs of cardiovascular disease. Forty percent of the invited (*n* = 314) agreed to participate. None had signs of diabetes nephropathy, retinopathy or neuropathy. Details from this study have been published earlier [22]. A total of 146 patients were examined by echocardiography as described previously [6], and included in the present study.

### Clinical and laboratory measurements

The baseline measurements of weight and height were recorded, and the body mass index (BMI) was calculated as the ratio of the individual’s body weight to the square of the height. Tanner stages of puberty were obtained from the Norwegian Childhood Diabetes Registry along with self-rating [22]. Arterial blood pressure (BP) was measured according to the National High Blood Pressure Education Program Working Group on High Blood Pressure in Children and Adolescents [23].

Glycated hemoglobin (HbA1C) was determined for all participants by liquid chromatography (Variant; Bio-Rad, Richmond, CA). The intra-assay coefficient of variation was <3%. Annual HbA1C values were available from the NCDR since 2001 or from the time of diagnosis for the majority of the patients. Mean HbA1C was calculated from all the HbA1C values available for each patient.

MG-H1 was measured by dissociation-enhanced lanthanide fluorescent immunoassay (DELFIA) as previously described [24, 25]. The applied anti-hydroimidazolone antibodies showed high sensitivity and little cross-reactivity. The assay was significantly and positively correlated with a stable isotopic dilution analysis liquid chromatography-mass spectrometry (LC-MS/MS) technique. (Unpublished observation by Dr. V. Monnier, Cleveland, OH, USA.)

### Glycemic burden

The glycemic burden (A1 months) was calculated using a modified version of the formula applied by Orchard et al. [26]. The first number of A1 months is the sum of months from the diagnosis of diabetes until the first HbA1c value registered in the NCDR multiplied by HbA1c units above the upper normal reference value (6.4%) of the first registered value. The same procedure is followed for every subsequent HbA1c measurement until the time of inclusion in the study. The sum of all the calculated A1 months represents the glycemic burden.

### Echocardiography

Tissue Doppler images (TDI) of left ventricle (LV) and right ventricle (RV) were measured from standard apical two and four chamber and long axis position as previously described in detail [6]. EchoPac software (EchoPac PC SW, GE, Horten, Norway) was used for post processing of the TDI indices. Early peak diastolic velocity (E’), atrial late peak velocity (A’), E’/A’-ratio and systolic peak velocity were analyzed from the lateral, septal, anterior and posterior mitral annulus. Peak systolic velocity (TDI S’) was measured at the maximum height of the systolic velocity curve.

With color tissue Doppler imaging (cTDI) we have previously demonstrated that 25% of children with T1D have E’/A’-ratio < 2.0, thus showing echocardiographic signs of reduced diastolic function, compared to only 10% of healthy control subjects [6]. In the present study, the patients were stratified accordingly.

### Common carotid artery ultrasound

A standard ultrasonic protocol was used for measuring the common carotid artery intima-media thickness (cIMT) as previously described in detail [22]. The distensibility coefficient (DC) as a measure of elasticity was calculated from the expression (DC = (ΔA/A)/ΔP = ((d + Δd)2-d2)/d2/Δp). Δp represents the change in pulse pressure, d the end-diastolic diameter (including the intima-media complex) and Δd the change in diameter from diastole to systole, assuming a circular lumen cross-section of the vessel. Young’s modulus, which reflects the elastic properties of the artery, was then calculated from these measurements as well as blood pressure assessment by a standard oscillometric device over the brachial artery [27].

### Statistical analysis

The T1D children were categorized into two groups according to their E’/A’-ratio. The 25% with the lowest E’/A’-ratio were categorized in a low diastolic function group (E’/A’-ratio < 2.0) and the rest of them in a reference group (E’/A’-ratio > 2.0). The data are presented as either means with their standard deviations or medians with 25th and 75th percentiles or proportions. Differences between the two groups were tested with Student’s t-test when normally distributed data, otherwise with Mann-Whitney U-test.

To identify possible independent baseline risk factors for E’/A’-ratio, univariate logistic regression analysis was employed. A significance level of 20% was deemed necessary for a variable to be included in the multivariate regression model. Subsequently, a manual backward stepwise elimination procedure was performed. The effect sizes were quantified by odds ratio (OR) with its 95% confidence interval. The predictive accuracy of the models was assessed by calibration and discrimination. Calibration was evaluated by the Hosmer and Lemeshow goodness-of-fit test. A statistically non-significant Hosmer and Lemeshow result (*p* > 0.05) suggests that the model predicts accurately on average. Discrimination was evaluated by analysis of the area under the ROC curve. We defined acceptable discriminatory capability as an area under the ROC curve greater than 0.7.

## Results

The clinical and metabolic characteristics of the participants are presented in Table 1. The patients categorized as the low diastolic function group had higher serum levels of MG-H1 and also echocardiographic signs of increased pressure in the left atrium with a higher E/E’ ratio than the control group. Furthermore, they had higher BMI, systolic blood pressure and diastolic blood pressure, as well as signs of increased arterial stiffness with a lower DC and higher Young’s modulus. There were no differences between the groups in years from diagnosis, actual HbA1c, mean HbA1c, estimated glycemic burden or CRP.

**Table 1:** Clinical and metabolic characteristics

	E’A’ < 2.0	E’A’ > 2.0	*p* - value
	*n* = 36	*n* = 110	
Age, years	14.5 (2.2)	13.3 (2.9)	0.008
Girls, n (%)	21 (58.3)	50 (45.5)	0.249
Diabetes duration, years^a^	5.3 (3.5, 6.2)	5.1 (2.5, 8.0)	0.701
Body Mass Index, kg/m^2^	22.8 (4.0)	20.1 (3.4)	<0 .001
Systolic blood pressure, mmHg	104.2 (8.7)	99.7 (9.3)	0.010
Diastolic blood pressure, mmHg	63.6 (8.3)	59.9 (7.9)	0.016
CRP mg/l^a^	0.59 (0.26, 2.08)	0.40 (0.27, 1.27)	0.348
Actual HbA1c, %	8.3 (1.0)	8.4 (1.4)	0.516
Mean HbA1c, %	7.9 (0.8)	8.1 (1.0)	0.194
Glycemic burden, A1 months	103 (96)	122 (104)	0.354
MG-H1, U/ml	163.9 (39.2)	150.3 (33.4)	0.046
E/E’	8.6 (2.0)	7.9 (1.5)	0.017
cIMT, mm	0.45 (0.05)	0.45 (0.05)	0.901
DC, kPa^−1^	0.035 (0.010)	0.042 (0.015)	0.013
Young’s modulus, kPa	429 (116)	365 (143)	0.009
Urine albumin/creatinine, mg/mmol^a^	0.6 (0.2, 1.2)	0.8 (0.4, 1.4)	0.248

Mean values (SD)

^a^Median (25th and 75th percentile)

The final logistic regression model is presented in Table 2. Only BMI was a significant risk factor for reduced diastolic function. Although MG-H1, which has a rather wide 95% confidence interval, did not reach significance as a risk factor for E’/A’-ratio, we included it in the model since the effect size, OR _per 50 U/ml increase_ = 1.52, suggested clinical importance. A 50 U/ml increase in MG-H1 corresponds to moving from the 25th to the 75th percentile. The Hosmer and Lemeshow goodness-of-fit test was not significant, indicating a satisfactory fit of the model (c2 = 8.49, df = 8, *p* = 0.39). The area under the ROC curve was 0.71 (95% CI: 0.62–0.81) indicating a good discriminative ability between patients with high and low E’/A’-ratio.

**Table 2:** Independent risk factors for E’/A’-ratio using logistic regression analysis

Dependent variable	Beta-value	Standard error	Odds ratio	95% Confidence Interval	*p*-value
MG-H1 per 50 U/ml	0.42	0.29	1.52	0.86–2.69	0.15
Body mass index per kg/m^2^	0.19	0.06	1.21	1.08–1.35	0.001

## Discussion

Early signs of reduced diastolic function in children and adolescents with T1D were associated with higher BMI. This is in harmony with a study by Suys et al., with a similar patient population as in ours, which showed increased BMI in the diabetes group as well as a correlation between BMI-SDS and diastolic function assessed by Tei-index, albeit only in boys [28]. Thus, these studies support a stronger emphasis on prevention of overweight and obesity than current guidelines suggest [29, 30].

Reduced aortic distensibility has been shown to be associated with reduced diastolic function in diabetic children and adults [31, 32]. We found significantly reduced carotid distensibility in the group of patients with impaired diastolic function as well as increased blood pressure. However, no measures of arterial stiffness were independent risk factors in our multivariate model.

It has been suggested that AGEs have a detrimental effect on ventricular function through either the formation of cross-links between collagen molecules or activation of the receptor for AGEs, RAGE, or both [11]. Previous studies indicate that MG-H1 can both create cross-links and activate RAGE, although this is still a disputed matter [33–36]. Glucosepane seems to be the most important cross-linking AGE, and it is not formed by the same pathway as MG-H1 [37]. This implies that even though MG-H1 may not be directly involved in the etiology of diabetic cardiomyopathy, it reflects an increased production of AGEs, some of which also may affect the heart. We found increased serum levels of MG-H1 in the diabetes patients with reduced diastolic function, but in the multivariate logistic regression model MG-H1 was not a significant independent risk factor. We believe this might be due to lack of statistical power, since the effect size shown by the odds ratio suggests a clinically relevant impact. In another study, we observed a significant association between E/E’, 2 D strain and serum MG-H1 in long term type 1 diabetes without significant coronary disease [13].

The relationship between diastolic dysfunction and HbA1c is not clear in children with T1D. Of the several studies of diastolic function in pediatric diabetes patients compared with healthy controls, some report a significant association with HbA1c [28, 38–40], while others do not [29, 41]. Our present study has a larger number of participants than all the comparable previous studies, and it also has better assessment of HbA1c. With data from the Norwegian Childhood Diabetes Registry, we have several HbA1c registrations, all performed at the same laboratory, in many patients as far back as the time of diagnosis. Thus, we have been able to calculate the glycemic burden over many years, strengthening the reliability of the data. We found no associations between diastolic function and years from diagnosis, HbA1c, mean HbA1c or calculated glycemic burden. This is surprising for two reasons. First, in addition to the referred pediatric studies [28, 39–41], a prospective study in young adults shows a convincing impact of HbA1c [42]. Secondly, given that AGEs play an important role in the development of diabetic cardiomyopathy, an association with HbA1c, itself an amadori product and a potential AGE predecessor, would be expected. The lack of association between metabolic control and reduced diastolic function in this study could indicate that the early reduced diastolic function in children with diabetes is partly caused by factors presented before the start of insulin treatment, and not reflected by HbA1c measured later. This needs further investigation.

The strengths of this study include a relatively large number of participants and longitudinal registrations of HbA1c. There are, however, also some weaknesses. Young’s modulus, reflecting the elasticity of an arterial wall, requires blood pressure measurements from the same artery and that the pulse pressure forces all work in one direction. Pubertal stage was included in the multivariate analysis, but puberty might still affect the results in an unpredictable fashion.

## Conclusions

Early signs of reduced diastolic function in children and adolescents with T1D were associated with higher BMI, but not higher HbA1c. They also had elevated serum levels of the advanced glycation end product MG-H1, higher blood pressure and increased stiffness of the common carotid artery, but these associations did not reach statistical significance when tested in a logistic regression model.
